# Clinical exhibition of increased accommodative loads for binocular fusion in patients with basic intermittent exotropia

**DOI:** 10.1186/s12886-016-0260-y

**Published:** 2016-06-07

**Authors:** Suk-Gyu Ha, Sung-Min Jang, Yoonae A. Cho, Seung-Hyun Kim, Jong-Suk Song, Young-Woo Suh

**Affiliations:** Department of Ophthalmology, Korea University Guro Hospital, 148, Gurodong-ro, Guro-gu, Seoul, 08308 South Korea; Department of Ophthalmology, Korea University Ansan Hospital, 123, Jeokgeum-ro, Danwon-gu, Ansan-si, Gyeonggi-do, 15355 South Korea; Nune Eye Hospital, 404, Seolleung-ro, Gangnam-gu, Seoul, 06198 South Korea; Department of Ophthalmology, Korea University Anam Hospital, 73, Inchon-ro, Seongbuk-gu, Seoul, 02841 South Korea

**Keywords:** Accommodation, Accommodative load, Intermittent exotropia, Open-filed autorefractor

## Abstract

**Background:**

To investigate the accommodative loads change needed to maintain binocular fusion in patients with intermittent exotropia (IXT).

**Methods:**

Seventeen consecutive patients with basic IXT and 15 normal controls were recruited. The WAM-5500 autorefractor (GrandSeiko, Fukuyama, Japan) was used to measure refractive error (D) under binocular and monocular viewing conditions at 6 m, 50 cm, 33 cm and 20 cm. The difference between binocular and monocular refractive error (D) at each distance defined the change in the accommodative load. The changes in accommodative load were compared between IXT patients and normal controls. We also investigated the change in accommodative loads according to the fixing preference in patients with IXT.

**Results:**

In IXT patients, the mean angles of deviation were 20.2 ± 7.19 and 21.0 ± 8.02 prism diopters at 6 m and 33 cm, respectively. Under binocular viewing, the changes in accommodative loads of each eye in IXT patients were significantly higher at 50, 33 and 20 cm than those of normal controls (*p* < 0.05, all). The changes in accommodative loads of fixating and deviating eyes at 6 m were not significantly different between IXT patients and normal controls (*p* = 0.193, 0.155, respectively). The changes in accommodative loads of the fixating eye at each distance were not significantly different from those of the deviating eye in IXT patients (*p* > 0.05).

**Conclusion:**

The changes of accommodative loads at near fixation increased more in IXT patients than they did in normal controls while maintaining binocular fusion.

## Background

Intermittent exotropia (IXT) is the most common form of childhood exotropia [1, 2] and is more prevalent in Asian children [3]. Although esotropia is associated with hyperopia and anisometropia, [4–6] the refractive error in patients with intermittent exotropia has not been extensively studied. A previous American population-based study found that 135 children with IXT showed a significant trend toward myopia over time [7]. Myopia is particularly prevalent in Asian populations; interestingly, exodeviation is at least twice as common as is esodeviation in Asia [8, 9]. The current literature is unable to prove that IXT may be causative for myopia. However, we hypothesize that there is a significant association between them, and that IXT may be a risk factor for myopic progression. Additional investigations are needed to clarify the relationship between IXT and myopia.

In the presence of IXT, binocular fusion requires more effort from the vergence system to produce additional convergence. Maintaining binocular motor fusion requires convergence to compensate for IXT. Since accommodation and convergence control are linked process [10, 11], this additional convergence likely evokes an accommodative response. Prolonged accommodation and near work are recognized as important causes of myopia [12, 13]. Therefore, this mechanism may explain the high prevalence of myopia in the IXT population. However, further research is needed to explain the increased accommodation required to maintain motor fusion in IXT.

The WAM-5500 (GrandSeiko, Fukuyama, Japan) is a binocular open-field autorefractor and keratometer that can dynamically record refractive error during binocular target fixation. The instrument uses an open-view window through which patients can see a target binocularly. Patients maintain binocularity during the examination because the refractive error can be measured without requiring any visual obstructions. Therefore, the examiner measures the refractive error of both eyes under binocular or monocular viewing conditions.

The purpose of this study was to investigate the increased accommodative loads required to maintain fusion in patients with IXT, and to compare the changes of accommodative loads with normal controls.

## Methods

Patients diagnosed with basic IXT between 5 and 20 years old and normal controls were enrolled. The study protocol was approved by the Korea University Medical Center Institutional Review Board. Written informed consent and the study were approved by the Research Ethics Committee at the Korea University Medical Center. This study adheres to the tenets of the Declaration of Helsinki. Written informed consent was obtained from all patients or parents after explanation of the study. All subjects underwent a complete ophthalmologic examination performed by a pediatric ophthalmologist (Y.W.S). Cycloplegic refraction was performed with cyclopentolate eyedrops when subjects required glasses. During the examination, subjects wore fully corrective glasses to achieve their best-corrected visual acuity (of 20/20 or better) in both eyes. The angle of deviation (prism diopters, PD) was determined using the prism and alternative cover test at short (33 cm) and long (6 m) distances. After monocular occlusion for an hour, the angle of deviation at the short distance was rechecked with and without an additional +3.00 D to evaluate the tenacious proximal fusion and accommodative convergence/accommodation ratio (AC/A). Patients were diagnosed with basic-type IXT if they met the following criteria: 1) normal accommodative convergence/accommodation ratio (as determined by the gradient method); 2) difference between long and short deviation <10 prism diopters; 3) lack of tenacious proximal fusion, which is widely used to differentiate pseudo-divergence excess from true divergence excess type of IXT, and is defined as the increase in near deviation after prolonged monocular occlusion over than 1 h [14]; 4) lack of strong proximal convergence after monocular occlusion for 1 h. Exclusion criteria included the following: amblyopia, anisometropia more than two diopters (D) in spherical equivalent (SE), refractive error exceeding −6.00 D or +3.00 D, astigmatism of >1.50 D, dissociated vertical deviation, vertical deviation, oblique muscle dysfunction, history of strabismus surgery, paralytic or restrictive exotropia, poor cooperation, ocular disease other than strabismus and systemic disorders. Patients with constant exotropia (at 6 m or 33 cm) who could not induce and maintain binocular fusion at each target distance were also excluded.

The WAM-5500 autorefractor/keratometer (GrandSeiko, Fukuyama, Japan) was used to measure refractive error under binocular and monocular conditions while subjects wore distant vision corrective lenses and fixated at 6 m, 50 cm, 33 cm and 20 cm. All measurements were performed by one of authors, an experienced ophthalmologist (S.M.J), confirming the maintenance of ocular alignment to orthotropia during measurements in the binocular viewing condition. And the measurements were also confirmed by another experienced ophthalmologist (S.G.H) to avoid bias which could be induced by a single examiner. Refractive error was presented as SE. At each distance, the increase in the accommodative load (according to the viewing condition) was calculated as the difference in the refractive errors between binocular and monocular viewing conditions.

The changes in the accommodative loads were compared between IXT patients and normal controls. The fixating and deviating eyes of IXT patients were also compared with the right and left eyes of control patients, respectively. The fixation preference in patients with intermittent exotropia was either determined using a cover-uncover test alternatively on each eye (at least three times), or by measuring the suppression at distance fixation using a Vectogram (Vectographic projector test, Reneau, France). If the fixation preference alternated, or was difficult to determine, the right and left eyes were defined as the fixating and deviating eyes for statistical analyses, respectively. The accommodative loads of the fixating eye were compared with those of the deviating eye in IXT patients.

Statistical analyses were performed using SPSS for Window version 20.0 (SPSS Inc., Chicago, IL, USA). The Mann–Whitney *U* test and Chi-square test were used to compare the baseline characteristics. The Wilcoxon-singed rank test and Kruskal-Wallis test were used to compare the accommodative load in the same eye and that between the fixating and deviating eyes. P values <0.05 were considered statistically significant.

## Results

A total of 17 patients and 15 normal controls were included in this study. The mean age was 10.0 ± 4.09 years (range, 5 to 20) in the IXT patients and 9.1 ± 2.58 years (range, 6 to 14) in the controls. Eight (53.1 %) IXT patients and 8 (46.9 %) controls were female. The mean angle of deviation in the IXT patients was 20.2 ± 7.19 PD (range, 12 to 35). Table 1 displays the basic patient characteristics.

**Table 1:** Baseline patient characteristics

	IXT (*n* = 17)	Controls (*n* = 15)	*p*
Mean age ± SD (range), yrs	10.0 ± 4.09 (5 to 20)	9.1 ± 2.58 (6 to 14)	0.711^*^
Female sex, no. (%)	8 (53.1)	8 (46.9)	0.723^**^
Mean refractive error ± SD (range), D			
Fixating eye	−0.74^a^ ± 1.42 (−4.75 to 1.00)	−1.27^a^ ± 2.07 (−5.25 to 0.88)	0.551^*^
Deviating eye	−0.60^a^ ± 1.44 (−4.88 to 1.13)	−0.92^a^ ± 1.88 (−5.75 to 0.75)	0.852^*^
Mean angle of deviation ± SD (range), PD			
Distance	20.2 ± 7.19 (12 to 35)	-	-
Near	21.0 ± 8.02 (12 to 40)	-	-

Refractive error is presented as spherical equivalent. Fixating and deviating eyes in patient with IXT were compared to right and left eyes in controls, respectively

*IXT* intermittent exotropia, *SD* standard deviation, *D* diopters, *PD* prism diopters

^*^ Mann–Whitney test, ^**^ Chi-square test

^a^ Negative values refer to myopic refractive error

In the fixating eye of IXT patients, the amount of accommodative load was significantly greater under binocular viewing than it was under monocular viewing during near target fixation (50, 33 and 20 cm) (*p* = 0.028, 0.001, 0.006 respectively). However, the accommodative loads during distant target viewing did not increase significantly (*p* = 0.193). Similarly, in the deviating eye of IXT patients, the amount of accommodative load under binocular viewing was also significantly greater than was that under monocular viewing during near target fixation (50, 33 and 20 cm) (*p* = 0.022, 0.003, 0.003 respectively). There were no significant changes in the accommodative loads during distant target viewing (*p* = 0.155). Tables 2 and 3 compare the refractive errors between monocular and binocular viewing conditions in IXT patients. The changes of accommodative loads for both fixating and deviating eyes increased as the target got closer (*p* = 0.047, 0.026, Kruskal-Wallis test).

**Table 2:** Accommodative load in the fixating eye of IXT patients

Target distance	Accommodative load		Difference ^a^, D	*p* ^*^
	Monocular fixation, D	Binocular fixation, D		
6 m	−0.27 ± 0.77 (−1.50 to 1.13)	−0.49 ± 0.54 (−1.56 to 0.87)	0.23 ± 0.58 (−0.51 to 1.88)	0.193
50 cm	−1.14 ± 0.63 (−3.00 to 0.00)	−1.55 ± 1.04 (−3.88 to 0.93)	0.42 ± 0.73 (−1.55 to 1.38)	0.028
33 cm	−1.68 ± 0.58 (−2.88 to −0.81)	−2.55 ± 0.92 (−5.44 to −1.56)	0.88 ± 0.74 (−0.07 to 2.56)	0.001
20 cm	−3.62 ± 0.77 (−5.18 to −2.12)	−4.50 ± 1.1 (−7.68 to −2.68)	0.89 ± 1.05 (−0.44 to 2.81)	0.006

*D* diopters

^*^ Wilcoxon’s signed rank test

^a^ Positive values indicate an increase in accommodative load with binocular viewing compared to monocular viewing

**Table 3:** Accommodative load in the deviating eye of IXT patients

Target distance	Accommodative load		Difference ^a^, D	*p* ^*^
	Monocular fixation, D	Binocular fixation, D		
6 m	−0.20 ± 0.60 (−1.13 to 0.88)	−0.49 ± 0.54 (−1.56 to 0.87)	0.20 ± 0.58 (−0.82 to 1.12)	0.155
50 cm	−1.13 ± 0.79 (−3.88 to −0.37)	−1.55 ± 1.04 (−3.88 to 0.93)	0.37 ± 0.77 (−1.74 to 1.44)	0.022
33 cm	−1.72 ± 0.68 (−2.93 to −0.76)	−2.55 ± 0.92 (−5.44 to −1.56)	0.67 ± 0.68 (−0.63 to 0.62)	0.003
20 cm	−3.20 ± 1.09 (−4.87 to −1.06)	−4.50 ± 1.11 (−7.68 to −2.68)	0.97 ± 1.08 (−1.63 to 3.07)	0.003

*D* diopters

^*^ Wilcoxon’s signed rank test

^a^ Positive values indicate an increase in accommodative load with binocular viewing compared to monocular viewing

However, there was no significant difference in the changes of accommodative loads with distance in normal controls (6 m, 50 cm, 33 cm and 20 cm) (*p* > 0.05, all). In addition, there were no significant differences between the right and left eyes in control patients. The changes in accommodative loads were also not significantly different according to the target distance (Tables 4 and 5).

**Table 4:** Accommodative load in the right eye of normal controls

Target distance	Accommodative load		Difference^a^, D	*p* ^*^
	Monocular fixation, D	Binocular fixation, D		
6 m	−0.42 ± 0.76 (−2.13 to 0.38)	−0.33 ± 0.79 (−2.50 to 0.81)	0.09 ± 0.28 (−0.69 to 0.37)	0.254
50 cm	−1.24 ± 0.64 (−2.63 to −0.38)	−1.27 ± 0.49 (−2.63 to −0.75)	0.03 ± 0.36 (0.82 to 0.44)	0.432
33 cm	−2.00 ± 0.61 (−2.81 to −0.69)	−2.10 ± 0.49 (−2.94 to −1.37)	0.10 ± 0.38 (−0.50 to 0.69)	0.348
20 cm	−3.80 ± 0.78 (−5.13 to 2.50)	−3.82 ± 0.87 (−4.93 to −2.13)	0.03 ± 0.89 (−1.38 to 1.75)	0.932

*D* diopters

^*^ Wilcoxon’s signed rank test

^a^ Positive values indicate an increase in accommodative load with binocular viewing compared to monocular viewing

**Table 5:** Accommodative load in the left eye of normal controls

Target distance	Accommodative load		Difference ^a^, D	*p* ^*^
	Monocular fixation, D	Binocular fixation, D		
6 m	−0.27 ± 1.04 (−2.69 to 1.31)	−0.30 ± 0.92 (−2.68 to 1.06)	0.03 ± 0.38 (−0.56 to 1.06)	0.969
50 cm	−1.23 ± 0.77 (−2.69 to 0.63)	−1.27 ± 0.78 (−2.62 to 0.25)	0.04 ± 0.47 (−1.07 to 0.70)	0.514
33 cm	−1.97 ± 0.79 (−2.87 to −0.06)	−1.97 ± 0.77 (−3.06 to −0.38)	0.00 ± 0.45 (−0.94 to 0.69)	0.733
20 cm	−3.58 ± 1.19 (−4.62 to 0.19)	−3.56 ± 0.98 (−5.13 to −1.25)	−0.03 ± 0.78 (−1.25 to 1.45)	0.776

*D* diopters

^*^Wilcoxon’s signed rank test

^a^ Positive values indicate an increase in accommodative load with binocular viewing compared to monocular viewing

While viewing distant targets, there were no significant differences in accommodative load change between the fixating eye of IXT patients and the right eye of control patients (6 m) (*p* = 0.097). However, while viewing near targets (50, 33 and 20 cm), there were significantly greater changes in accommodative loads in the fixating eyes of IXT patients than there were of right eyes from controls (*p* = 0.027, 0.002, 0.002, respectively). These results are similar to the changes in accommodative loads of the deviating eyes in IXT patients compared to those of left eyes from controls. During distant target (6 m) viewing, there were no significant differences in accommodative load change between the deviating eyes of IXT patients and the left eyes from controls (*p* = 0.278). However, during near target viewing (33 and 20 cm), there were significantly greater changes of accommodative loads in the deviating eye of IXT patients than there were of left eyes from controls (*p* = 0.002, 0.003). Table 6 compares the changes of accommodative loads between IXT patients and controls.

**Table 6:** Comparing increases in accommodative load from monocular to binocular viewing between IXT patients and controls

Target distance	Fixating/right eye of IXT patients, D	right eye of controls, D	*p* ^*^	Deviating eye/left of IXT patients, D	left eye of controls, D	*p* ^*^
6 m	0.23 ± 0.58 (−0.51 to 1.88)	0.09 ± 0.28 (−0.69 to 0.37)	0.097	0.20 ± 0.58 (−0.82 to 1.12)	0.03 ± 0.38 (−0.56 to 1.06)	0.278
50 cm	0.42 ± 0.73 (−1.55 to 1.38)	0.03 ± 0.36 (0.82 to 0.44)	0.027	0.37 ± 0.77 (−1.74 to 1.44)	0.04 ± 0.47 (−1.07 to 0.70)	0.064
33 cm	0.88 ± 0.74 (−0.07 to 2.56)	0.10 ± 0.38 (−0.50 to 0.69)	0.002	0.67 ± 0.68 (−0.63 to 0.62)	0.00 ± 0.45 (−0.94 to 0.69)	0.002
20 cm	0.89 ± 1.05 (−0.44 to 2.81)	0.03 ± 0.89 (−1.38 to 1.75)	0.022	0.97 ± 1.08 (−1.63 to 3.07)	−0.03 ± 0.78 (−1.25 to 1.45)	0.003

*IXT* intermittent exotropia, *D* diopters

^*^Mann–Whitney test

Among IXT patients with a confirmed fixation preference, there were no significant differences in accommodative load change between fixating and deviating eyes at each target (*p* > 0.05, all, Table 7).

**Table 7:** Comparing the increase in accommodative load from monocular to binocular viewing between the fixating and deviating eyes of IXT patients with fixation preferences

Target distance	Fixating eye, D	Deviating eye, D	*p* ^*^
6 m	0.23 ± 0.58 (−0.51 to 1.88)	0.20 ± 0.58 (−0.82 to 1.12)	0.653
50 cm	0.42 ± 0.73 (−1.55 to 1.38)	0.37 ± 0.77 (−1.74 to 1.44)	0.368
33 cm	0.88 ± 0.74 (−0.07 to 2.56)	0.67 ± 0.68 (−0.63 to 0.62)	0.356
20 cm	0.89 ± 1.05 (−0.44 to 2.81)	0.97 ± 1.08 (−1.63 to 3.07)	0.435

*IXT* intermittent exotropia, *D* diopters

^*^Mann–Whitney test

## Discussion

There is an increased change in accommodative loads in IXT patients during binocular viewing of a near target. This finding demonstrates that, when focusing on a near target, IXT patients require excessive accommodation to maintain single binocular vision (range, 0.37 to 0.97 D). In addition, the accommodative loads of patients with IXT rise with decreasing fixating distance during binocular viewing.

The accommodative load of fixating and deviating eyes in IXT were compared right and left eyes of normal control respectively. It would be more informative if we could compare fixating and deviating eyes in IXT with dominant and non-dominant eyes of normal control. However, the control group included young children, and some of them could not understand our question during dominant eye test. So we could not determine the ocular dominance in the normal control. When we compare accommodative response between right and left eyes of normal controls, there were no difference. So we compared fixating and deviating eyes with right and left eye respectively.

In a population-based study of children, Ekdawi et al. reported that, by 20 years of age, >90 % of patients with IXT exhibit myopia [7]. However, others have found that the initial refractive error in IXT children is not much different from that of control patients. In 62 exotropic children with a mean age of 4.4 years, Kushner found an average refractive error of plano [15]. Caltrider et al. reported a mean refractive error of −0.669 D in 15 children with a mean age of 6.9 years [16]. These studies suggest that a large number of myopic patients with IXT may develop myopia faster than do healthy peers without IXT.

Many prior studies have attributed myopia to excessive accommodation [17–19]. Hasebe et al. reported that, in heterophoria, there are different accommodation responses under monocular and binocular conditions [20]. The group demonstrated that the fusional demand to compensate for heterophoria affects the accommodative response via AC/A linkage. This interaction between convergence and accommodation plays an important role for near vision. When focusing on a close target, IXT patients require more convergence to achieve single binocular vision than do people without IXT. This increased convergence would induce more accommodation. Our study shows that there is a 0.97 D increase in accommodation from monocular to binocular viewing. This increase in accommodation can be one of supporting factors for the suggested hypothesis that IXT patients develop and progress myopia faster than normal population.

This study has several limitations. The sample size was relatively small, and may not adequately represent the changes in accommodative load in all IXT patients. In addition, the included patients were only diagnosed with the basic type of IXT. The other types of IXT, including convergence insufficiency and divergence excess, may have influenced the results differently given their complicated mechanisms of accommodation. Therefore, results in this study may not be generalized to all types of IXT. Furthermore, cycloplegic refraction was not performed for subjects who did not require glasses. However, the purpose of this study was to focus on differences between each measurement according to distance, and to determine accommodation increases in daily life. Therefore, cycloplegic refraction was not performed in every subject. We cannot exclude the possibility that subjects may have been accommodating more with distant fixation and have variably abnormal accommodation with near fixation; however, this is not likely.

## Conclusions

The accommodative loads of IXT patients increased most when they fixated on close objects with binocular, single vision. This finding may contribute to the high prevalence of myopia in IXT patients. Further studies are needed to determine whether myopic progression is related to changes in the accommodative load in IXT patients, and whether this can be addressed surgically.

## Abbreviations

D, diopters; IXT, intermittent exotropia; PD, prism diopters; SD, standard deviation; SE, spherical equivalent
